# The Effects of Sex and Body Weight on Renal Graft Function—The Role of CCL2

**DOI:** 10.3390/jcm10214951

**Published:** 2021-10-26

**Authors:** Magdalena Nalewajska, Martyna Opara-Bajerowicz, Krzysztof Safranow, Andrzej Pawlik, Kazimierz Ciechanowski, Sebastian Kwiatkowski, Ewa Kwiatkowska

**Affiliations:** 1 Department of Nephrology, Transplantology and Internal Medicine, Pomeranian Medical University, 70-111 Szczecin, Poland; nalewajska@gmail.com (M.N.); martyna.opara@wp.pl (M.O.-B.); kazcie@pum.edu.pl (K.C.); 2 Department of Biochemistry and Medical Chemistry, Pomeranian Medical University, 70-111 Szczecin, Poland; krzysztof.safranow@pum.edu.pl; 3 Department of Physiology, Pomeranian Medical University, 70-111 Szczecin, Poland; pawand@poczta.onet.pl; 4 Department of Obstetrics and Gynecology, Pomeranian Medical University, 70-111 Szczecin, Poland; kwiatkowskiseba@gmail.com

**Keywords:** chemokines, CCL2, kidney transplantation, weight excess, sex dimorphism

## Abstract

There are reports on the effects of excessive recipient body weight on renal graft function. Increased CCL2 (chemokine CC-mortif ligand 2) production is observed in patients with excessive body weight. CCL2 also exacerbates the inflammatory process in the renal graft. A total of 49 renal graft recipients of both sexes having undergone renal biopsy within the last 18 months were retrospectively reviewed. At their most recent appointment the patients’ plasma concentrations of CCL2 were evaluated. Renal function was assessed retrospectively. CCL2 concentrations were higher in men than women (*p* < 0.047), while higher CCL2 levels were associated with a decrease in eGFR (estimated glomerular filtration rate) during the first year post Tx (kidney transplantation). CCL2 negatively correlated with eGFR at 5 years (R = −0.45, *p* < 0.040997) and positively correlated with the degree of tubular atrophy in renal biopsy specimens (R = 0.43, *p* < 0.027293) and with systolic pressure. Men showed significantly higher BMI (body mass index) values at the time of Tx and at their last appointment than women did (*p* < 0.000403; *p* < 0.000613, respectively). Men showed poorer long-term renal graft function, with significantly lower eGFR values at 4 and 5 years into the post-transplantation period. The male sex and excessive body weight have adverse effects on short- and long-term renal graft function, which is associated with increased levels of CCL2.

## 1. Introduction

Kidney transplantation (Tx) is the best acknowledged renal replacement therapy [1]. The treatment of patients in the initial post-transplantation period is well controlled due to the availability of new immunosuppressive drugs, improved surgical techniques and better perioperative care [2]. However, contemporary transplantology is still facing challenges related to preventing, diagnosing and treating progressive chronic graft failure [2]. Interstitial fibrosis and tubular atrophy (IF/TA) found in renal biopsy specimens is one of the main causes of progressive renal graft dysfunction and is responsible for approximately 30% of its cases [3]. The development of fibrous lesions in the histopathological picture of the renal graft, and the resulting poorer graft function, may be contributed to by ischemia-reperfusion (IRI) lesions and the accompanying inflammation [4]. It is believed that IRI may be associated with delayed renal graft function and poorer graft function in the short- and long-term follow-up period after Tx [3,4]. This is linked to the activation of oxidative stress, the generation of reactive oxygen species and neutrophil recruitment [4].

Chemokines are small proteins from the cytokine group which, by binding with a specific receptor, play the role of chemoattractants and allow immune cells to migrate towards the inflammation site [5]. Chemokines include proinflammatory chemokines, such as CCL2 (chemokine CC-mortif ligand 2), which may contribute to the intensification of inflammatory responses in various tissues, including in the renal graft, thereby leading to the development of chronic lesions and the resulting chronic graft dysfunction [2]. In previous studies, CCL2 levels positively correlated with IF/TA processes in kidney grafts by promoting the accumulation and activation of immune cells in renal interstitium and by increasing the production of pro-fibrotic factors that further leads to the occurrence of fibrotic changes in the kidney [2].

Increased CCL2 production is observed in, inter alia, patients with excessive body weight and obesity [5,6]. Excessive body weight is commonly defined as BMI (body mass index) from 25.0 up to 29.9 kg/m^2^, whereas obesity is defined as BMI 30.0 kg/m^2^ and higher. Some hormones, e.g., androgens, also contribute to increasing CCL2 concentrations [7]. Patients with polycystic ovary syndrome (PCOS), a condition characterized by, inter alia, hyperandrogenemia and obesity, show higher CCL2 levels than those found in healthy persons [7]. Adipocytes are capable of increased CCL2 production leading to the recruitment of macrophages from the circulatory system into the adipose tissue and a further production of proinflammatory cytokines, thus driving the inflammatory cascade, which results in the development of metabolic complications [6]. Excessive body weight is also considered to affect the occurrence of inflammation in the renal tissue, which may lead to the impairment of renal function and the development of chronic kidney disease [8]. We therefore hypothesized that excessive body weight and associated increased CCL2 production could also apply to worsened short- and long-term kidney graft survival. We conducted the present study to evaluate how body weight and sex affect renal graft function and to examine the role that CCL2 plays in these processes.

## 2. Results

The concentration of CCL2 determined at the most recent appointment (ME, median, 70 months after Tx) was statistically significantly higher in men than women (*p* < 0.047), as shown in Table 1. CCL2 levels correlated negatively with ΔeGFR (delta eGFR; estimated glomerular filtration rate) at 1 year, i.e., the higher the CCL2 concentration the lower the eGFR value at 1 year in relation to ZENITH eGFR (the highest estimated glomerular filtration rate within the first 6 months post renal transplantation ). CCL2 concentrations correlated negatively with eGFR at 5 years (R = −0.45, *p* < 0.040997), positively with the degree of IF/TA determined in renal biopsy specimens (R = 0.43, *p* < 0.027293) and positively with systolic pressure.

Higher CCL2 concentrations were found in patients with post-transplantation diabetes mellitus, although no statistical significance (*p* = 0.08) was reached, which may have resulted from the small size of the study group. Men had significantly higher BMI values at the time of Tx and at the most recent appointment than women did (*p* < 0.05), as shown in Table 1. Despite the comparable function of the renal graft at 1 year in terms of ZENITH GFR and GFR, men had poorer long-term renal graft function with significantly lower eGFR at 4 and 5 years into the follow-up period. Additionally, men demonstrated more frequent DGF (delayed graft function) and acute graft rejection (AR) throughout the follow-up period. Patients with high BMIs at Tx showed a higher incidence of DGF (*p* < 0.05), as shown in Table 2. The BMI at Tx correlated positively with the presence of urine protein (*p* < 0.05, R 0.33).

## 3. Discussion

### 3.1. CCL2/MCP-1

Chemokines are a group of chemotactic cytokines participating in inflammatory cascade regulation, cell proliferation and mast cell, basophil and fibroblast activation [9]. CCL2, formerly referred to as MCP-1 (monocyte chemoattractant protein-1), is one of the inflammatory chemokines that are responsible for monocyte/macrophage, T lymphocyte and natural killer cell migration into the inflammation site and that participate in the production of CD8+ memory T cells [10]. Chemokines are produced by multiple cell types, e.g., macrophages, lymphocytes, neutrophils, vascular endothelial cells, fibroblasts and keratinocytes [9]. In the kidney, chemokines are produced by renal tubular and glomerular epithelial cells [10]. It is currently believed that chemokines, including CCL2, may also be produced by adipocytes of the adipose tissue. CCL2 has recently been introduced as one of main causative factors for the development of renal fibrosis by promoting the accumulation of immune factors in kidney interstitium and releasing profibrotic factors, therefore leading to decreased graft survival [2]. Excessive CCL2 expression associated with obesity may also contribute to the development of inflammation in the renal tubulointerstitial tissue [8]. Therefore, CCL2 seems to be the potential factor linking these phenomena with each other.

### 3.2. CCL2/BMI

In the present study, male kidney recipients showed significantly higher serum CCL2 concentrations at sampling than women did. This phenomenon might be related to the larger volume of the adipose tissue in men than women (the BMI at Tx and at sampling was significantly higher in men than women). Many reports link obesity with increased CCL2 production, although none discuss kidney recipients. Excessive weight is associated with the presence of the systemic low-key inflammatory state [5]. Adipocytes and macrophages linked to the adipose tissue are responsible for the production of proinflammatory cytokines and chemokines. CCL2 produced by adipocytes promotes recruitment of monocytes from blood and their maturation to adipose tissue macrophages (ATMs), which then leads to the production of proinflammatory molecules and further drives the inflammatory cascade and the related metabolic complications [6]. Plasma CCL2 concentrations are higher in the obese adults and children [11]. The visceral adipose tissue (VAT) stimulates inflammation to a higher extent that the subcutaneous adipose tissue (SAT) does [12]. Adipose tissue distribution depends on, for instance, sex and sex steroids [12]. In women, SAT prevails, while men demonstrate prevalence of VAT [12]. VAT predisposes men to produce more CCL2 than in the case of women. A strong correlation has been shown between the expression of CCL2 and TNFα (tumor necrosis factor alpha) in VAT, but not in SAT [13]. In a study performed by Azizian et al., a significant relationship was found between waist circumference and plasma MCP-1 levels [14]. In another study, the plasma concentration of MCP-1 was higher in patients with higher contents of VAT [15].

Sex, and more specifically androgens, are also associated with increased CCL2 levels. Numerous reports have attempted to explain this phenomenon, but no research has been carried out among kidney recipients. The role of androgens in developing adipose tissue and promoting inflammation is inconclusive. Some authors have implicated that androgens participate in the development of VAT and the formation of insulin resistance [16,17]. Testosterone is believed to be responsible for the increased volume of VAT in men as compared to women [17]. Furthermore, postmenopausal women show a growing content of VAT in line with an increase in testosterone concentrations [17]. The expression of genes coding for, inter alia, CCL2 is higher in VAT than SAT, and that expression is higher in male VAT than female VAT [18]. In the same study, female mice treated with testosterone were observed to show upregulated expression of CCL2 in VAT and an increased VAT content [18]. Barbosa-Desongles et al. showed in their study that testosterone caused an increase in the numbers of preadipocytes in VAT [17]. They claimed that this mechanism could be responsible not only for the predominance of VAT in men as compared to women, but also contribute to the central location of the adipose tissue in hyperandrogenic women.

Most research into the effects of androgens on the development of adipose tissue and inflammation is being carried out on groups of female patients with PCOS characterized by hyperandrogenemia and the prevalence of the visceral adipose tissue. In a study performed by Crisosto et al., obese patients with PCOS demonstrated upregulated CCL2 expression in VAT when compared to non-obese women, and the expression correlated positively with the BMI [19]. Incubation of samples collected from VAT with testosterone led to increased CCL2 expression in these tissues, and the expression also correlated positively with the BMI [19]. The authors of that paper suggested that the effects of testosterone and obesity on the development of inflammation in VAT were synergistic.

In the present study, patients with higher plasma CCL2 levels had a tendency to develop post-transplantation diabetes mellitus, although no statistical significance (*p* = 0.08) was shown in this case, which may have resulted from the small size of the study group. It is believed that CCL2 may contribute to the development of insulin resistance (IR) [19]. Sartipy et al. made an in vitro observation of reduced insulin sensitivity of murine mature adipocytes incubated even with low MCP-1 concentrations [20]. On the other hand, MCP-1 inhibition in mice reduced insulin resistance [21]. Furthermore, the G-2518A polymorphism of the *CCL2* gene has been shown to be associated with the occurrence of IR [22]. In the same study, patients with IR were shown to have higher CCL2 concentrations than those without IR [22]. In a study carried out by Dabrowska-Zamojcin et al., the *CCL2* rs1024611 polymorphism was shown to be associated with the development of post-transplantation diabetes mellitus in patients receiving calcineurin inhibitors [9].

### 3.3. CCL2/Short- and Long-Term Renal Graft Function

Short-term renal graft function describes starting the function of the transplanted kidney (delayed versus immediate graft function) and graft function during the first year after kidney transplantation. DGF is defined as the need for hemodialysis within the first 7 days after kidney transplantation and is one of the most common post-transplant complications. Acute kidney injury secondary to DFG may be linked with poorer short- and long-term renal graft function, or even complete graft loss [23]. Acute tubular necrosis (ATN) is the most common cause of DGF [23]. Some factors that may lead to the development of ATN are reported to be: epithelial and endothelial damage due to occlusion of the tubules, microvascular flow impairment, and immunological and inflammation processes [23]. In the present study, patients with high BMI values at Tx were characterized by a higher incidence of DGF. Similar results have previously been obtained. A meta-analysis performed in 2015 showed that obesity was associated with a 68% increase in the odds of DGF [24]. Another meta-analysis performed in 2016 showed that DGF was 81% more prevalent in obese patients than those with a normal BMI [25].

The mechanism responsible for poorer renal graft function in obese patients has not been fully explained yet and appears to be multifactorial. Obese patients are believed to demonstrate increased hyperfiltration and exacerbated proteinuria, which are independent risk factors for progressive glomerulosclerosis and thus lead to reduced GFR values [24]. Similar results were obtained in the present study: the patients’ BMI at Tx correlated positively with the presence of urine protein at the time of examination. Moreover, as an organ playing an immuno-endocrine role, the adipose tissue makes an additional contribution to the worsening of renal function by producing inflammatory molecules [24,25]. In the present study, it was shown that the adipose tissue and CCL2 that it produces had an adverse effect both on short- and long-term renal graft function. Plasma CCL2 concentrations at sampling correlated negatively with ΔeGFR at 1 year after Tx in relation to ZENITH eGFR. This means that patients with higher CCL2 concentration on their last visit had greater reduction of eGFR during first year after transplantation. We assume that high concentration of CCL2 is already present in the perioperative period and remains high for the rest of the time after renal transplantation. Furthermore, eGFR determined in the study carried out by Liese et al. at 12 months after Tx was significantly lower in obese patients than otherwise [26]. Similar results were obtained by Kieszek et al. and Erturk et al., as well as Kanthawar et al. in patients with extreme obesity (a BMI > 50 kg/m^2^) [27,28,29]. In our study, only two patients displayed BMI ≥ 30 kg/m^2^, whereas other patients with excessive body weight had BMI in the range between 25.0 and 29.9 kg/m^2^. None of the patients included in the research had morbid obesity (defined as BMI of ≥35 kg/m^2^ and experiencing obesity-related health conditions or ≥40–44.9 kg/m^2^); people with highly increased BMI are not commonly indicated as transplant recipient candidates as perioperative complications in this group are more frequent. As morbid obesity counts for a different condition, therefore our results could preferably be applied only to patients with excessive body weight or secondly with first stage obesity.

Moreover, in the present study men that had significantly higher plasma CCL2 levels and higher BMIs had poorer renal graft function parameters determined by way of GFR estimated using the MDRD formula than women in long-term follow-up at 4 and 5 years after Tx. Additionally, ZENITH eGFR during first six months was the same in the groups of women and men. CCL2 negatively correlated with eGFR at 5 years (R = −0.45, *p* < 0.040997) and positively correlated with the degree of IF/TA in renal biopsy specimens (R = 0.43, *p* < 0.027293) and with systolic pressure. Infiltration of the renal tissue by inflammatory cells, such as monocytes, may promote the development the renal graft inflammation and fibrosis [2]. To a large extent, infiltration is made worse by CCL2, mainly the type produced locally in the kidney [2]. In a study performed by Yan et al., higher expression of MCP-1 and RANTES (Regulated on Activation, Normal T-cell Expressed and Secreted) in the allograft tissue was associated with the occurrence of interstitial fibrosis and tubular atrophy, and correlated with the severity of IF/TA [2]. In another study, the urine concentration of CCL2 at 6 months after Tx was an independent risk factor for the development of IF/TA and inflammation in the renal graft [30]. As for the urine CCL2:Cr (CCL2 to creatinine) ratio, this has been found to be an early marker of long-term renal graft function and graft loss; low CCL2:Cr values are correlated with graft survival [10]. Apart from exacerbating the inflammatory cascade, macrophages recruited into the renal tissue with the participation of chemokines are believed to contribute to the increased production of fibrotic factors, e.g., TGF-β1 (transforming growth factor β1) and PDGF (platelet-derived growth factor), which elevate the production of extracellular matrix and type IV collagen and exacerbate renal fibrosis, which could explain the higher risk of renal function deterioration and graft loss in this group of kidney recipients [2]. Research has shown that infiltration by CD68+ macrophages in protocol graft biopsy specimens with IF/TA and inflammatory lesions at 1 year after Tx correlated with the severity of graft dysfunction and the odds of graft loss, and infiltration with CD68+ and CD206+ macrophages correlated with the severity of IF/TA and graft dysfunction at 3 years after Tx [31,32].

## 4. Materials and Methods

### 4.1. Patients

A total of 49 renal graft recipients treated by the Clinical Department of Nephrology, Transplantology and Internal Medicine, Pomeranian Medical University, Szczecin, Poland were retrospectively reviewed. The study included patients who reported within a two-week period in 2019 for a standard follow-up appointment. As inclusion criteria, the patients were expected to have received their kidney transplant at least 1 year before, and to have had a renal biopsy specimen collected within the last 12 to 18 months. The patients were after Tx for an average of 70 months (ME). After Tx, all the patients received triple immunosuppressive therapy with glucocorticosteroids, a calcineurin inhibitor, tacrolimus or cyclosporin, and mycophenolate mofetil. In some of the patients the steroid therapy was discontinued during the long-term follow-up. The parameters that were tested were the highest value of eGFR (ZENITH GFR) achieved within the first 6 months post Tx, creatinine concentration and eGFR at the most recent appointment (when plasma was being sampled for CCL2 determinations), and creatinine concentration and eGFR at 1, 2, 3, 4, 5 and 10 years post Tx. The GFR was estimated using the MDRD formula. ΔeGFR, or the changes of eGFR over time in relation to ZENITH eGFR, was also evaluated. The occurrence of DGF, defined as the need for hemodialysis within the first week after transplantation, was evaluated. Urinalysis was carried out with a focus on the presence of urine protein (mg/dL). At the most recent appointment, the plasma concentration of CCL2 was evaluated. The renal biopsy specimens were examined for irregularities such as interstitial inflammation (I), tubulitis (T), glomerulitis (G), peritubular capillaritis (PTC), the presence of C4D, interstitial fibrosis (IF), tubular atrophy (TA), vascular fibrous intimal thickening (CV), double contours of the GBM (CG), mesangial matrix expansion (MM), arteriolar hyalinosis (AH) and hyaline arteriolar thickening (AAH). Recipient characteristics such as age, sex, time from Tx, the BMI at the time of Tx and at the most recent appointment, PRA (panel-reactive antibody), CIT (cold ischemia time) and the numbers of mismatches for different types of HLAs (human leukocyte antigen) were evaluated. An estimation was made for the occurrence of new-onset diabetes after transplantation (NODAT), which was diagnosed according to the American Diabetes Association (ADA) criteria (2017) involving the following: the symptoms of diabetes (i.e., polyuria, polydipsia or unexplained weight loss) plus random plasma glucose of ≥200 mg/dL; fasting plasma glucose (FPG) of ≥126 mg/dL, with fasting defined as no caloric intake for at least 8 h; and 2 h plasma glucose of ≥200 mg/dL during an oral glucose tolerance test (OGTT). Donor characteristics, such as age, sex, creatinine concentration, eGFR and serum sodium concentration prior to organ procurement, were also evaluated, and are presented in Table 3. Patient details are presented in Table 4.

### 4.2. Methods

Plasma samples were centrifuged at 4000 rpm for 10 min, and sediment-free plasma was stored at a temperature of −80 °C awaiting analysis. CCL2 concentration was assessed according to the manufacturer’s instructions using customized magnetic bead-based multiplex Luminex screening immunoassay kits (R&D Systems). The patients involved in the study were treated in line with the Declaration of Helsinki and the Declaration of Istanbul. The local ethics committee of the Pomeranian Medical University, Szczecin, Poland approved the study protocol KB-0012/23/18 (05FEB2018) and all participants gave their written informed consent. All of the participants received organs from deceased donors that were over 18 years old. According to Polish law, if a deceased person did not express objection while alive (in the Central Objection Register, in written form or in the presence of at least two witnesses and confirmed by them in writing), recovering tissues or organs from such deceased persons for transplantation purposes is allowed. All of the participants received organs from donors that did not express any objection while being alive.

### 4.3. Statistical Analysis

We used Statistica 11 software (StatSoft, Tulsa, OK, USA) for statistical analysis. The Shapiro–Wilk test was used to study the distribution. The distribution of CCL2 was significantly different from normal (*p* < 0.05). We used a nonparametric Mann–Whitney U test to compare the two groups. Spearman’s rank correlation test was used to study correlations. Data that were not normally distributed were shown as the median [minimum-maximum]. *p*-values were considered significant if <0.05.

## 5. Conclusions

In conclusion, our study reports that both adipose tissue and male sex had adverse effects on both short- and long-term renal graft function. We postulate that CCL2, a chemoattractant chemokine responsible for renal graft inflammation and fibrosis produced by adipocytes, among others, may be a key link between those two phenomena, as we concluded that BMI was larger and CCL2 serum concentrations were increased in male kidney recipients compared to females. However, due to the small sample size, further investigation needs to be performed in order to verify these findings.

## Figures and Tables

**Table 1:** Clinical characteristics of the female (W) and male (M) groups.

Recipients	W (*n* = 23)			M (*n* = 26)			*p*
	ME	SD	MEAN	ME	SD	MEAN	
Age	43.1	12.4	41	48	13.6	49.8	0.027332
BMI at Tx	21.4	2.9	22	25.8	3.9	26.3	0.000403
BMI at last appointment	22.9	3.4	23.6	27.7	4.1	27.8	0.000613
Time from Tx (months)	87.2	56.1	92	54	44.9	61	NS
Duration of dialysis prior to Tx (months)	14	26.5	25.6	14.5	21.7	22.46	NS
PRA	3	18.9	11.46	0	13.9	6.22	NS
CIT (min)	1170	667.4	1139	1108	446.7	1095.5	NS
Mismatch							NS
A	1	1	0.8	1	0.7	0.7	
B	1	0.7	0.8	1	0.64	0.7	
DR	1	0.5	0.94	1.1	0.64	1.1	
ZENITH eGFR	57.5	18.5	62	58.06	16.11	58.5	NS
eGFR at 1 year	48.2	19.5	51.1	43.2	16.5	45.2	NS
eGFR at 2 years	48.9	17.9	51.1	45.3	16.4	45.8	NS
eGFR at 3 years	48.2	12.8	51.6	44.2	16.7	44	NS
eGFR at 4 years	50.9	19.8	56	42.32	14.84	40.4	0.0248
eGFR at 5 years	57.47	21.4	58.6	36.9	10.22	34.5	0.000677
CCL2 (pg/mL)	166	93.7	192	228.6	87	233	0.047
	%						
AF	4/23 (17.4%)			7/26 (27%)			0.049
DGF	5/23(22%)			7/26 (27%)			0.049

BMI—body mass index; Tx—kidney transplantation; PRA—panel reactive antibody; CIT—cold ischemia time; DGF—delayed graft function; eGFR—estimated glomerular filtration rate (estimated using the MDRD formula); ZENITH eGFR—the highest estimated glomerular filtration rate within the first 6 months post renal transplantation (estimated using the MDRD formula); NS—not statistically significant; *p*—*p* value; AR—acute rejection; ME—median; N—sample size; SD—standard deviation.

**Table 2:** BMI at Tx values in DGF POSITIVE and DGF NEGATIVE patients.

	DGF POSITIVE					DGF NEGATIVE					
	ME	MIN	MAX	MEAN	SD	ME	MIN	MAX	MEAN	SD	*p*
BMI at Tx	24.9	20.1	34.8	26.2	4	22.7	16.3	31.2	23.5	3.85	0.039

BMI—body mass index; Tx—kidney transplantation; DGF—delayed graft function; min—minimum; max—maximum; ME-median; SD—standard deviation; *p*—*p* value.

**Table 3:** Clinical kidney donor characteristics for both groups (male/female).

Donor Characteristics	W			M			*p*
	ME	MEAN	SD	ME	MEAN	SD	
Age	41	43.7	15	49	49.7	11	NS
BMI	23.4	24.1	3.2	23.5	25.8	3.7	NS
eGFR (mL/min/1.73 m^2^)	72	91.45	6.1	75	84.9	40	NS
Sodium concentration (mmol/L)	146.5	149.6	9.34	146	148.9	10.74	NS

ME—median; SD—standard deviation; *p*—*p* value; BMI—body mass index; eGFR—estimated glomerular filtration rate; W—femaile; M—male; NS—not statistically significant.

**Table 4:** Clinical characteristics of the study group.

	N	ME	MIN	MAX	MEAN	SD
Time from Tx (months)	49	69	12	182	75.7	52.3
Recipient age (years)	49	42	24	71	45.7	13.6
BMI at Tx	49	23.8	16.1	34.8	24.4	4
BMI at last appointment	49	25.4	18.1	34	25.9	4.3
eGFR at last appointment (mL/min/1.73 m^2^)	49	36	15	89	38.6	19.1
Urine protein (mg/dL)	49	0	0	865.7	78.7	165
Duration of dialysis prior to Tx (months)	49	15	0	102	23.9	23.8
CIT (min)	49	1260	72	2100	1116.7	555
PRA (%)	49	3	0	56	8	15.9
ZENITH eGFR (mL/min/1.73 m^2^)	49	31.0	98.0	118.0	58	17
eGFR at 1 year (mL/min/1.73 m^2^)	49	45	9	96.0	47.7	17.9
eGFR at 2 years (mL/min/1.73 m^2^)	48	49	15	85	48	17.1
eGFR at 3 years (mL/min/1.73 m^2^)	43	47	17	87	47.3	15.4
eGFR at 4 years (mL/min/1.73 m^2^)	38	46	19	98	48	18.9
eGFR at 5 years (mL/min/1.73 m^2^)	35	41	17	109	45.5	20.1
eGFR at 10 years (mL/min/1.73 m^2^)	13	44	23	82	47.8	18.7
CCL2 (pg/mL)	49	204.2	41.8	438.2	213.9	91.6
sex	49 (23 W, 26 M)					

N—sample size; ME—median; MIN—minimum; MAX—maximum; SD—standard deviation; Tx—renal transplantation; BMI—body mass index; eGFR—estimated glomerular filtration rate (estimated using the MDRD formula); ZENITH eGFR—the highest estimated glomerular filtration rate within the first 6 months post renal transplantation (estimated using the MDRD formula); W—female group; M—male group.
